# Assessment of the In Vivo Antioxidant Activity of an Anthocyanin-Rich Bilberry Extract Using the *Caenorhabditis elegans* Model

**DOI:** 10.3390/antiox9060509

**Published:** 2020-06-10

**Authors:** Ana M. González-Paramás, Virginia Brighenti, Laura Bertoni, Laura Marcelloni, Begoña Ayuda-Durán, Susana González-Manzano, Federica Pellati, Celestino Santos-Buelga

**Affiliations:** 1 Grupo de Investigación en Polifenoles, Unidad de Nutrición y Bromatología, Facultad de Farmacia, Universidad de Salamanca, Campus Miguel de Unamuno, 37007 Salamanca, Spain; paramas@usal.es (A.M.G.-P.); bego_ayuda@usal.es (B.A.-D.); susanagm@usal.es (S.G.-M.); 2 Department of Life Sciences, University of Modena and Reggio Emilia, Via G. Campi 103, 41125 Modena, Italy; virginia.brighenti@unimore.it (V.B.); laurabertoni21@gmail.com (L.B.); 186958@studenti.unimore.it (L.M.)

**Keywords:** *Vaccinium myrtillus* L., ROS, thermal stress, insulin/IGF-1 signaling, DAF-16, HSF-1

## Abstract

Anthocyanins have been associated with several health benefits, although the responsible mechanisms are not well established yet. In the present study, an anthocyanin-rich extract from bilberry (*Vaccinium myrtillus* L.) was tested in order to evaluate its capacity to modulate reactive oxygen species (ROS) production and resistance to thermally induced oxidative stress, using the nematode *Caenorhabditis elegans* as an in vivo model. The assays were carried out with the wild-type N2 strain and the mutant strains *daf-16(mu86)* I and *hsf-1(sy441)*, which were grown in the presence of two anthocyanin extract concentrations (5 and 10 μg/mL in the culture medium) and further subjected to thermal stress. The treatment with the anthocyanin extract at 5 μg/mL showed protective effects on the accumulation of ROS and increased thermal resistance in *C. elegans*, both in stressed and non-stressed young and aged worms. However, detrimental effects were observed in nematodes treated with 10 μg/mL, leading to a higher worm mortality rate compared to controls, which was interpreted as a hormetic response. These findings suggested that the effects of the bilberry extract on *C. elegans* might not rely on its direct antioxidant capacity, but other mechanisms could also be involved. Additional assays were performed in two mutant strains with loss-of-function for DAF-16 (abnormal DAuer Formation factor 16) and HSF-1 (Heat Shock Factor 1) transcription factors, which act downstream of the insulin/insulin like growth factor-1 (IGF-1) signaling pathway. The results indicated that the modulation of these factors could be behind the improvement in the resistance against thermal stress produced by bilberry anthocyanins in young individuals, whereas they do not totally explain the effects produced in worms in the post-reproductive development stage. Further experiments are needed to continue uncovering the mechanisms behind the biological effects of anthocyanins in living organisms, as well as to establish whether they fall within the hormesis concept.

## 1. Introduction

*Vaccinium* berries, and particularly bilberry (*Vaccinium myrtillus* L.), are considered to be one of the richest sources of anthocyanins, which have been related to several biological activities closely connected with their pharmacokinetic characteristics [1,2,3,4,5,6,7]. The anthocyanin composition may notably differ among different species of *Vaccinium*. In particular, *V. myrtillus* is characterised by the presence of 3-*O*-galactosides, 3-*O*-glucosides and 3-*O*-arabinosides from delphinidin, cyanidin, petunidin, peonidin and malvidin, while only delphinidin and cyanidin glycosides have been found in *V. floribundum* [6]. Anthocyanins are considered to possess one of the lowest bioavailability among the different polyphenol classes [8]. Being highly water-soluble molecules, they are hardly absorbed by passive diffusion and they require specific active transport mechanisms [8]. There is evidence that some anthocyanins could be absorbed in their native form in a small percentage in the stomach and small intestine [9,10,11]. After that, they are quickly metabolised, found in the blood, and excreted into bile and urine as intact compounds and phase II metabolites (methylated, glucuronide and/or sulphate conjugated forms). Studies in animal models and humans have indicated that they appear in the bloodstream within a few minutes (6 to 20 min) after consumption and that they reach maximum blood levels at 15 to 60 min [12,13,14]. Their apparent bioavailability has been estimated within a range of less than 1 to 2%, with only trace quantities detected in the bloodstream and expected target organs [15]. Studies performed with radiolabeled anthocyanins have shown that, despite anthocyanins being little bioavailable, their total bioavailability is greater when both the original compounds and all their metabolite pool are considered, that is, phase I and phase II metabolites and metabolites resulting from degradation by gut microbiota (e.g., benzoic, phenylacetic and phenylpropanoic acids, phenolic aldehydes, and hippuric acid) [13]. Actually, the most important anthocyanin metabolites correspond to products of microbial degradation and their related phase II conjugates, which may reach 60- and 45-fold higher concentrations than those of their parent compounds in the urine and plasma, respectively, reaching maximum levels at about 24 h after intake [13,14].

The consumption of bilberry fruits and extracts has been proposed to contribute to the prevention of several chronic diseases, such as cardiovascular diseases (CVDs), conditions associated with the metabolic syndrome (e.g., diabetes, hypertension and obesity) and some types of cancer [1,2,3,4,5,6,7], as well as to ameliorate neuronal and cognitive decline in ageing [16,17]. These beneficial effects have been most often attributed to their anthocyanin and polyphenol composition, largely demonstrated to possess noteworthy antioxidant and radical scavenging capacities, as well as ability to modulate the activity of transcription factors and gene expression in stress-related pathways [5,18,19].

Oxidative stress results from an imbalance between the production of reactive oxygen species (ROS) and the antioxidant defence system, which can generate important cell damage [20], which is assumed to be at the origin of the physiopathology of many chronic and age-related diseases [17,21,22,23,24]. ROS are constantly produced in the organism in the electron-transport chain in mitochondria, as a result of the activity of several enzymatic systems (e.g., cytochrome P450, NAD(P)H oxidases, lipoxygenases, etc.) and Fenton-like reactions and also being generated from exogenous sources like physical or chemical stressors. Even if a ROS excess is toxic, these species are necessary as cell signalling molecules, and they can also mediate the adaptive stress response of cells [25,26].

Different in vitro assays have been developed to measure the antioxidant activity of polyphenols extracted from different plant and food sources, including anthocyanins, the most common ones being oxygen radical absorbance capacity (ORAC), 2,2-diphenyl-1-picrylhydrazyl (DPPH), 2,2′-azinobis (3-ethyl-benzothiazoline-6-sulfonic acid) (ABTS) and ferric reducing ability of plasma (FRAP) assays [27,28]. Despite the wide use of in vitro assays, they differ from each other regarding their reaction mechanisms, oxidant and target/probe species, assay conditions and result expression. For that reason, in order to have a better idea of the antioxidant capacity, the use of different assays measuring different dimensions of antioxidant behaviour is usually recommended [29].

An alternative to the above-cited in vitro assays is represented by *Caenorhabditis elegans*. This nematode has been used as an in vivo model to study the effects and mechanisms of action of xenobiotics [30], such as of polyphenols, especially as related to their ability to modulate stress resistance and ageing [31,32,33,34,35,36,37,38].

Regarding the influence of anthocyanins on *C. elegans* lifespan and/or stress resistance, whereas some authors have reported positive effects [39,40,41,42], others have concluded that anthocyanins did not exert beneficial effects on the worm [43]. According to these latter, the active fraction in terms of the antioxidant capacity of blueberries would be rather represented by proanthocyanidins than by anthocyanins [43]. It is important to point out that the compositions of the anthocyanin extracts used in these studies are different in each case, as determined by the original plant material and extract preparation used, which could explain the distinct results obtained among them. Thus, in the assayed mulberry (*Morus alba* L.) [39], açai (*Euterpe precatoria* Mart.) [41] and pitanga (*Eugenia uniflora* L.) extracts [42], more than 80% of the anthocyanins were cyanidin derivatives (glucosides and rutinosides), while in purple wheat [40], approximately 80% were cyanidin and peonidin glucosides. In the only study dealing with blueberries (*Vaccinium angustifolium*) [43], no information on the composition was given. Furthermore, most of these extracts contained not only anthocyanins but also other phenolic and non-phenolic compounds.

Despite the controversial results, most authors have agreed on the ability of anthocyanins to modulate several signalling pathways, among them the evolutionary conserved insulin/insulin-like growth factor 1(IGF-1) signalling pathway (IIS), which is involved in relevant biological processes, such as development, reproduction, metabolism, somatic maintenance, or stress resistance [39,40,41,42]. The ability to activate DAF-16/FOXO, the main transcription factor involved in the regulation of stress-response genes in the IIS pathway, promoting its translocation to the nucleus and subsequent transcriptional activity, was proposed as a possible mechanism for anthocyanin action [40,41,42]. Nevertheless, in the study by Wilson et al. [43], the treatment of *daf-16* and *skn-1* knockout mutants of *C. elegans* with blueberry polyphenols led to prolonged lifespans, suggesting that these compounds may act independently of these genes.

The present study was aimed at the assessment of the in vivo antioxidant capacity of an anthocyanin-rich extract prepared from bilberry fruits harvested from three different areas of the Italian Northern Apennines by using *C. elegans* as a model organism. The study was carried out using the wild-type N2 strain and the loss-of-function mutant strains *daf-16(mu86)* I and *hsf-1(sy441)*, so as to try to elucidate the implication of these genes in the mechanism of action of anthocyanins.

## 2. Materials and Methods

### 2.1. Chemicals and Solvents

Sodium chloride (NaCl), calcium chloride (CaCl_2_) potassium phosphate monobasic (KH_2_PO_4_), potassium phosphate dibasic (K_2_HPO_4_), sodium hydroxide (NaOH), sodium phosphate dibasic (Na_2_HPO_4_), sodium hypochlorite (NaClO) solution 10% *w/v*, dimethyl sulfoxide (DMSO), acetonitrile (ACN), hydrochloric acid (HCl), methanol (MeOH), trifluoroacetic acid (TFA) and *tert*-butyl hydroperoxide (tBuOOH) were purchased from Panreac (Barcelona, Spain). Agar, cholesterol, sodium ampicillin, nystatin, bacto-yeast, 5-fluoro-2′-deoxyuridine (FUdR), phosphate buffered saline and 2’,7’-dichlorofluorescein diacetate (DFCH-DA) were purchased from Sigma-Aldrich (Madrid, Spain). Peptone enzymatic digest from soybean, bacto-tryptone and trichloroacetic acid (TCA) were provided by Fluka (Madrid, Spain). Magnesium sulphate, tween 20 (poly-oxy-ethyl-ensorbitan-monolaurate) and acetic acid were acquired from Merck (Darmstadt, Germany). Water (H_2_O) was purified by using a Milli-Q Plus185 system from Millipore (Milford, MA, USA). Reversed-phase C_18_ resin (12–20 µm) was purchased from Sigma-Aldrich.

### 2.2. Bilberry Fruit Samples

Bilberry fruits (*Vaccinium myrtillus* L.) were harvested from low bushes located in three different areas of the Italian Northern Apennines (i.e., Lago Scaffaiolo, Abetone and Fanano, in Modena province). For the preparation of the anthocyanin-rich extract, fruit samples belonging to the different geographic areas were mixed and stored at −20 °C until required.

### 2.3. Preparation of the Anthocyanin-Rich Extract from Bilberry Fruit

Extracts were prepared using widely used standardised procedures, as described elsewhere [44,45]. In brief, a portion of 75 g of frozen bilberry fruits was crumbled and placed into a flask with 200 mL of MeOH-2% HCl (95:5 (*v*/*v*)). The extraction mixture was put in an ultrasonic bath for 20 min at room temperature and subsequently centrifuged for 10 min at 6700× *g*. The mixture was then paper-filtered, and the extract was collected. The solid residue was submitted to the same extraction process further three times. The extracts were combined and concentrated under reduced pressure to remove MeOH. Previously, a few millilitres of ultrapure H_2_O were added to the extract to prevent an increasing acid concentration in the solvent mixture as the MeOH evaporated, which might have resulted in anthocyanin deglycosylation. The obtained aqueous extract was then purified using a C_18_ resin, which was suspended in acetone, placed in a Buchner funnel and conditioned by successive washing with H_2_O. The extract was laid on the resin layer on the funnel and washed with approximately 2 L of H_2_O, thus removing sugars and more polar substances. Then, MeOH-2% HCl (95:5 (*v/v*)) was added to elute the anthocyanins [46,47]. The acidic methanol extract rich in anthocyanins was concentrated under vacuum, as indicated above, and further freeze-dried. The composition of the extract was analysed by high-performance liquid chromatography coupled to diode-array detection (HPLC-DAD) and electrospray ionisation mass spectrometry (ESI-MS).

### 2.4. HPLC-DAD-MS Analysis of Anthocyanin-Rich Extract

The method used for anthocyanin analysis was based on methods previously established [48], slightly adapted to provide improved bilberry anthocyanin separation. One milligram of the lyophilised extract was dissolved in a known volume of ACN- 0.1% TFA (1:9 (*v/v*)) to be injected into the HPLC system. The analyses were performed in an Agilent Technology modular model 1200 system, equipped with a binary pump and a diode array detector (DAD). The chromatographic separation of the bioactive compounds was achieved with an AQUA^®^ C_18_ column (150 mm × 4.6 mm, 5 μm) (Phenomenex, Alcobendas, Spain), thermostated at 35 °C. The mobile phase was composed of (A) 0.1% TFA and (B) ACN. The gradient elution was set as follows: 0–3 min isocratic elution at 10% B, 3–15 min from 10% to 15% B, 15–20 min isocratic elution at 15% B, 20–25 min from 15% to 18% B, 25–45 min from 18% to 30% B, 45–50 min from 30% to 35% B. The flow-rate was set at 0.5 mL/min. The DAD acquisition wavelengths were at 280 and 520 nm. A mass spectrometer coupled to the HPLC system, via a DAD cell outlet, was also used for detection. In particular, MS detection was performed in the positive ion mode, using a hybrid triple quadrupole/linear ion trap API 3200 Qtrap^®^ (Applied Biosystems, Darmstadt, Germany), equipped with an ESI source. MS spectra were acquired from 100 *m/z* to 1500. Zero grade air was used as the nebuliser gas (40 psi) and as the turbo gas (600 °C) for solvent drying (50 psi). Nitrogen served as the curtain (100 psi) and collision gas (high). Both quadrupoles were operated at unit resolution, and the MS detector was set to perform a full scan of high sensitivity (enhanced MS, EMS) and an enhanced product ion analysis (EPI) to get the fragmentation pattern of the precursor ions. The EMS mode parameters were as follows: ion spray voltage, 5000 V; declustering potential (DP), 41 V; entrance potential (EP), 7.5 V; and collision energy (CE), 10 V. The EPI mode was performed under the following conditions: DP, 41 V; EP, 7.5 V; CE, 10 V; and collision energy spread (CES), 0 V. Anthocyanin identification was performed by comparison with standard compounds, when available, as well as on the basis of their chromatographic behaviour, UV/Vis and mass spectra in comparison with our library database and literature data. Quantification was performed from the areas of their peaks recorded at 520 nm, using a calibration curve prepared with a standard of delphinidin-3-*O*-glucoside.

### 2.5. Strains and Maintenance Conditions

Nematode handling and assays were carried out according to standardised procedures described in reference handbooks [49] and usually employed in our laboratory [33,34,37,38].

*Caenorhabditis elegans* wild-type N2 and the mutant strains *daf-16(mu86)* I and *hsf-1(sy441)* I as well as *Escherichia coli* OP50 were provided by the Caenorhabditis Genetics Centre of the University of Minnesota (Minneapolis, MN, USA). All *C. elegans* strains were propagated at 20 °C using nematode growth medium (NGM) plates with *E. coli* OP50 as the food source. The synchronisation of worm cultures was performed by treating gravid hermaphrodites with bleach-NaOH 5 N (2:1). The suspension was vigorously shaken for one minute, and then it was left to rest for a further minute. This procedure was repeated five times. The worms were dissolved in the bleach solution, while the eggs were resistant to bleach. The suspension was centrifuged (2 min, 9500× *g*), and the pellet containing the eggs was washed four times with an equal volume of buffer M9 (3 g of KH_2_PO_4_, 6 g of Na_2_HPO_4_, 5 g of NaCl, 1 mL of 1 M MgSO_4_, and H_2_O added to 1 L). After the removal of the supernatant, the eggs were re-suspended and they were kept in a small volume of M9. A volume from 100 to 200 μL of M9 with eggs (depending on egg concentration) was transferred to and incubated in NGM agar plates (Ø 100 mm), containing the anthocyanin extract, and cultivated at 20 °C. Control plates without the extract were simultaneously assayed. The lyophilised anthocyanin extract dissolved in DMSO was added to the nematode growth medium during its preparation to obtain final concentrations of 5 and 10 μg/mL in the plates. Control plates were prepared using the same volume of DMSO (0.1% DMSO *v/v*). Worms at the L4 stage were transferred to new plates, with or without the anthocyanin extract, but also containing 5-fluorodeoxyuridine (FUdR) at a concentration of 150 μM to prevent reproduction and progeny overgrowth. Every two days, worms were transferred to new plates, control or treatment plates, with FUdR until they reached the day of the assay (2nd or 9th of adulthood).

### 2.6. Stress Assays

This assay was carried out in agreement with the method described by Saul et al. [50]. Oxidative stress in worms was induced by their exposure to a temperature of 35 °C, which causes damage by ROS accumulation [51]. Worms were incubated at 20 °C on NGM–*E. coli* OP50 plates containing or not (control group) the anthocyanin extract (5 and 10 μg/mL) and FUdR 150 μM until days 2 and 9 of adulthood. At these days, nematodes were individually transferred to new control or treatment plates (Ø 35 mm, 20 worms per plate) to be subjected to thermal stress (35 °C, 8 h) in an incubator. Then, dead and alive nematodes were counted; in particular, worms were scored as dead when they did not respond to a touch stimulus with a platinum wire. The assays were carried out on about 100 nematodes per treatment, and each test was repeated at least in triplicate. The results were finally given as the percentage of living nematodes in each assay. The relative rates of worm survival after thermal stress were expressed in relation to the untreated control.

### 2.7. Accumulation of Reactive Oxygen Species (ROS)

The accumulation of ROS was evaluated at the end of the 2nd and 9th days of adulthood in the N2 strain cultivated in the presence and absence of the anthocyanin extract. Cellular ROS were quantified using the dichlorofluorescein assay with a microplate reader [52]. Briefly, each worm was transferred to the well of a 96-well plate, containing 75 μL of PBS, and then exposed to thermal stress (35 °C) for 2 h. Then, 25 μL of 150 μM DCFH-DA (2′,7′-dichlorodihydrofluorescein diacetate) solution in PBS buffer was added to each well. The acetate groups of DCFH-DA were removed in worm cells, and non-fluorescent DCFH was oxidised by intracellular ROS to produce the fluorescent dye DCF. The fluorescence from each well was evaluated at 35 °C immediately after the incorporation of the reagent and then every 10 min for 60 min, with a 1 s integration time, at 485 and 535 nm as the excitation and emission wavelengths, respectively. The DCF fluorescence intensity over time in single worms was measured, and it was used as an index of the individual intracellular ROS levels. Three independent experiments were carried out for each treatment, and, for each experiment, the ROS measurements were performed on at least 24 individual worms. These analyses were carried out using an ultra-evolution multi-functional microplate reader (Tecan, NC, USA).

### 2.8. Statistical Analyses

Statistical analyses were performed with the SPSS software. An ANOVA post-test was used to make multiple comparisons of mean values to establish possible significant differences between the treated and the control groups in the ROS assays, while contingency tables were prepared to analyse survival of thermal stress, and statistical significance was calculated using the Chi Square Test. The differences were considered statistically significant at *p* < 0.05.

## 3. Results and Discussion

### 3.1. Analysis of Anthocyanins in the Bilberry Extract

**Figure 1:**
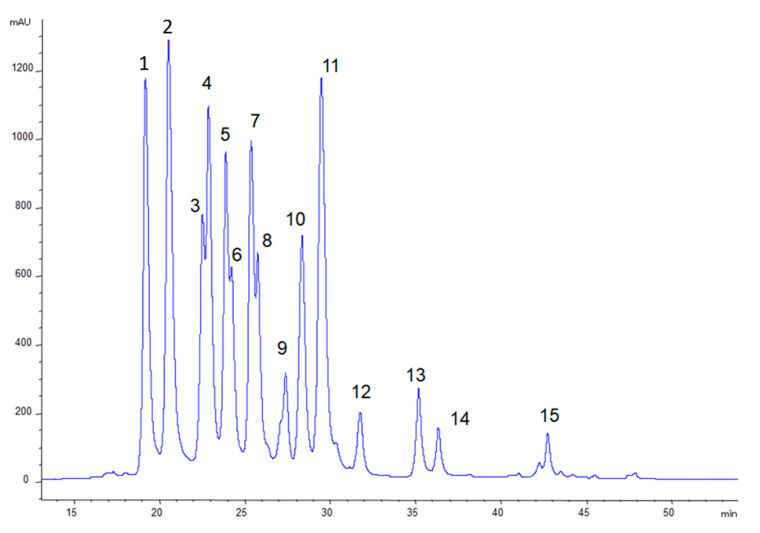
HPLC chromatogram recorded at 520 nm of the bilberry purified anthocyanin extract. For peak identification, see Table 1.

The anthocyanin-rich purified extract obtained as described in Section 2.4 was analysed by HPLC-DAD and ESI-MS. A representative chromatogram is shown in Figure 1.

The identity and content of anthocyanins in the bilberry extract are shown in Table 1. In total, 17 anthocyanins were detected, comprising glucosides, galactosides and arabinosides of delphinidin, cyanidin, petunidin, peonidin and malvidin, in agreement with what has been previously reported in the literature for the anthocyanin profile of bilberries [6]. Three anthocyanidins were also detected, probably resulting from the cleavage of sugar residues during the preparation of the anthocyanin extract. It must be noted that the composition of the prepared extract practically consisted of anthocyanins, as verified by the absence of other significant peaks when the chromatogram was recorded at 280 nm, at which all polyphenols absorb to a greater or lesser extent.

### 3.2. C. elegans Assays

Thermal stress is associated with oxidative damage caused by an increased production of intracellular ROS [51]. ROS accumulation in a time-dependent manner in *C. elegans* subjected to thermal stress was confirmed in different studies [31,35]. In the present work, the ability of bilberry anthocyanins to modulate ROS levels and improve thermotolerance was assessed in the wild-type *C. elegans* N2 strain.

**Figure 2:**
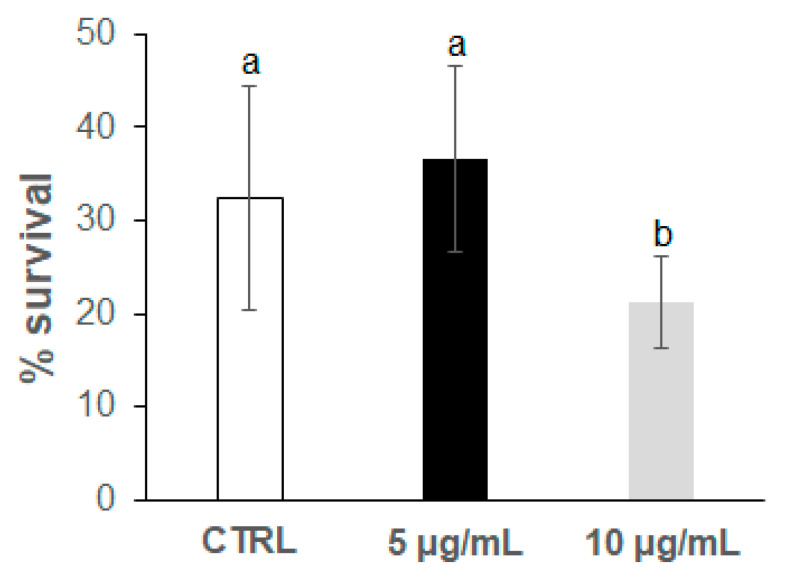
Percentages of worm survival at the 2nd day of adulthood after the application of thermal stress (35 °C, 8 h). The results were obtained in the *C. elegans* wild-type N2 strain grown in the absence (controls) and presence of the bilberry anthocyanin extract at concentrations of 5 and 10 μg/mL in the culture medium. The results are presented as the mean values ± SD. Different letters between bars indicate significant difference, *p* < 0.05.

**Figure 3:**
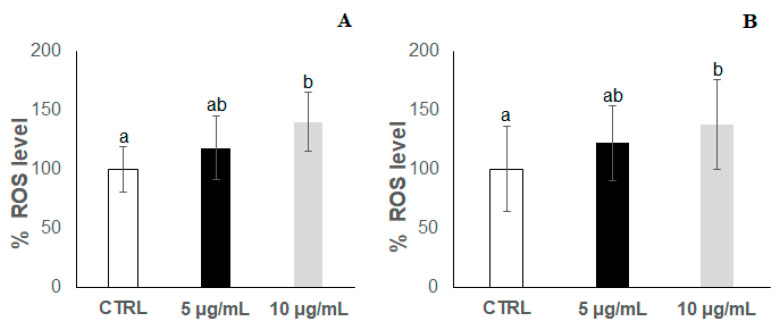
Reactive oxygen species (ROS) accumulation (expressed as percentage of fluorescence relative to controls) at the 2nd day of adulthood in *C. elegans* wild-type N2 strain grown in the absence (controls) and presence of the bilberry anthocyanin extract at concentrations of 5 and 10 μg/mL in the culture medium. Results obtained in (**A**) non-stressed worms and (**B**) worms subjected to thermal stress (35 °C, 2 h). In all cases, the assays were carried out in triplicate. The results are presented as the mean values ± SD. Different letters between bars indicate significant differences, *p* < 0.05.

**Figure 4:**
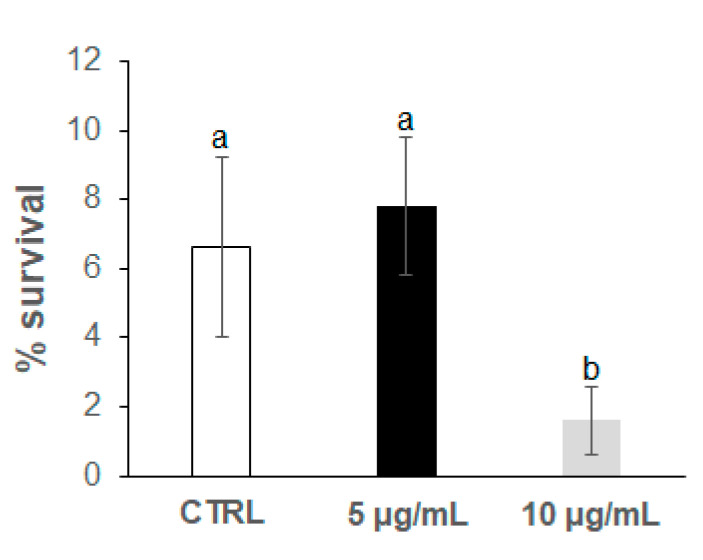
Percentages of worm survival at the 9th day of adulthood after the application of thermal stress (35 °C, 8 h). The results were obtained in the *C. elegans* wild-type N2 strain grown in the absence (controls) and presence of the bilberry anthocyanin extract at concentrations of 5 and 10 μg/mL in the culture medium. The results are presented as the mean values ± SD. Different letters between bars indicate significant differences, *p* < 0.05.

**Figure 5:**
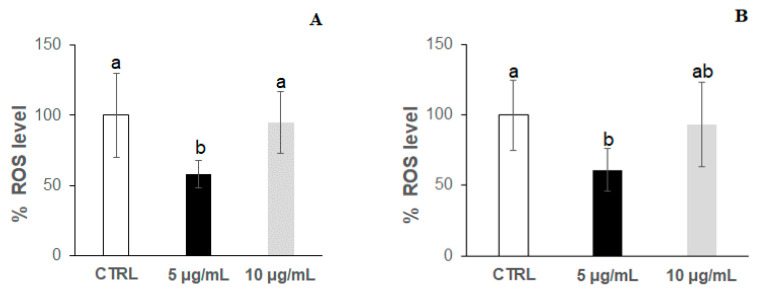
ROS accumulation (expressed as percentage of fluorescence relative to controls) at the 9th day of adulthood in *C. elegans* wild-type N2 strain grown in the absence (controls) and presence of the bilberry anthocyanin extract at concentrations of 5 and 10 μg/mL in the culture medium. Results obtained in (**A**) non-stressed worms and (**B**) worms submitted to thermal stress (35 °C, 2 h). In all cases, the assays were carried out in triplicate. The results are presented as the mean values ± SD. Different letters between bars indicate significant differences, *p* < 0.05.

In a previous screening, a range of concentrations up to 300 μg/mL of the anthocyanin extract was checked, finding that detrimental effects (high rate of total worm death) were produced in the *C. elegans* populations at concentrations higher than 25 μg/mL in the culture media. For this reason, lower concentrations of 5 μg/mL and 10 μg/mL were now assayed. Thermal stress (35 °C) was applied at the 2nd and 9th day of adulthood in worms grown in the presence of the bilberry extract at two different concentrations (5 and 10 μg/mL); the results were then compared with those from control worms grown in the absence of anthocyanins and subjected to the same assay conditions. The results obtained are shown in Figure 2, Figure 3, Figure 4 and Figure 5. In all cases, the results came from several independent experiments, performed at different periods of time on different synchronised populations, using a minimum of 100 worms per thermotolerance assay, and 24 individual worms in each assay for ROS levels. In every case, the experiments were carried out at least in triplicate.

Figure 2 shows the percentage of worm survival after the exposure to thermal stress at the 2nd day of adulthood in the distinct groups of assays. After thermal stress, a non-significant (*p* = 0.26) slight increase in thermotolerance was observed in the worms grown in the presence of 5 μg/mL of the bilberry extract, in relation to the control group. However, the nematodes treated with 10 μg/mL of the extract significantly decreased their resistance to thermal stress; so, this anthocyanin concentration not only did not protect worms against stress, but also showed detrimental effects.

Figure 3a,b show the results for ROS accumulation in worms at the 2nd day of adulthood without and with thermal stress, respectively. Interestingly, the ROS level significantly increased in the nematodes treated with 10 μg/mL of bilberry extract, regardless of the application of thermal stress. An increase in the level of ROS in the worms treated with 5 μg/mL of bilberry extract was also observed, although it was not significant compared to controls. These findings revealed that, far from leading to a ROS decrease, the anthocyanin extract at the assayed concentrations induced their accumulation. Actually, despite polyphenols usually acting as free radical scavengers and antioxidants, they may also have a pro-oxidant activity in in vivo conditions, depending on their concentration and the biological system [53], which might explain the apparent concentration-dependent increase found in the ROS levels.

Different observations were, however, made in the assays carried out at the 9th day of worm adulthood (Figure 4 and Figure 5). A greater decrease in the survival rate after thermal stress was found in the nematode populations (around 7% in control worms, Figure 4) compared to their younger counterparts (around 32% at the 2nd day of adulthood, Figure 2). This lower tolerance to stress might be attributed to their older age. Nevertheless, as for younger animals, a non-significant slight increase in survival was observed at the lower anthocyanin concentration (5 μg/mL), and a strong decrease, at the highest one (10 μg/mL), newly suggesting deleterious effects on worm thermotolerance at increased anthocyanin concentrations.

Regarding ROS accumulation, a similar behaviour was found at the 9th day in both non-stressed (Figure 5a) and stressed (Figure 5b) worms treated with the bilberry extract, with a significant decrease in ROS levels at the lower anthocyanin concentration (5 μg/mL) and a non-significant slight decrease at 10 μg/mL in relation to controls. This behaviour totally differs from that observed in younger nematodes (2nd day of adulthood), where the treatment with the bilberry extract at either concentration always resulted in an increase in ROS levels (Figure 3). The significant decrease in ROS levels observed in the worms grown in the presence of 5 μg/mL of bilberry extract might be consistent with the slightly higher (though not significant) survival rate in this group of nematodes (Figure 4). However, in the worms treated at 10 μg/mL, the apparent ability to prevent a ROS increase (as produced in younger animals) does not reflect in an improved survival, but rather a dramatic decrease was produced. Meng et al. [54] have observed that, in response to paraquat, *C. elegans* young individuals demonstrated a stronger ability to generate ROS and they argued that the ability to generate ROS is a capacity that serves as a response to stress, activating signalling pathways to maintain redox homeostasis. This could also explain our results for the differential effects of the treatment with the bilberry extract on both ROS production and *C. elegans* survival, depending on the specific worm life stage.

Taken as a whole, the findings obtained in this work seem to point out that, whereas low anthocyanin concentrations may improve worm resistance against thermal stress, an increase of their concentration above certain levels can be detrimental, suggesting a hormetic response. Moreover, this response does not seem to be related to their ability to modulate ROS levels, otherwise unclear, and differs depending on worm age, being more pronounced in older animals. Similar findings have already been described in previous studies of ours and other groups, where hormetic responses against stress in *C. elegans* treated with different flavonoids have been observed, not strictly related to ROS levels [37,55,56].

Unlike our results, other authors have observed a significant increase in the survival rate of *C. elegans* treated with different anthocyanin-rich extracts and subjected to oxidative stress. Peixoto et al. [41] found that, under juglone-induced oxidative stress, the survival rate of young worms treated with an anthocyanin-rich extract of açai (*Euterpe precatoria* Mart), at a concentration of 200 μg/mL in the culture medium, was significantly higher when compared to that of the untreated control group (up to 86% vs. 39%). Tambara et al. [42] studied the effects of an ethanolic extract of purple pitanga (*Eugenia uniflora* L.), rich in cyanidin-3-*O*-glucoside, but also containing other anthocyanins, flavonols and gallic acid derivatives. They found that this extract, at a range of concentrations from 5 to 500 μg chlorogenic acid equivalent (CAE)/mL in the medium, was able to improve the survival of worms subjected to different oxidative stress situations (juglone, H_2_O_2_ or thermal stress), as well as to decrease ROS production and extend the lifespan in the wild-type N2 strain and *mev-1* mutants. These effects were not observed to the same extent for all the concentrations assayed, but they were especially evident at the highest concentrations. By contrast, Wilson et al. [43], in a study performed on wild-type N2 and different knockout *C. elegans* strains, did not find any protective effect of blueberry polyphenols (200 μg/mL in the culture medium) against hydrogen peroxide-induced oxidative stress; however, the same extract was able to increase survival in worms subjected to acute heat stress (35 °C for 16 h). Nonetheless, from the different major components of their assayed extract (proanthocyanidins, anthocyanins and hydroxycinnamic esters), only the proanthocyanidin fraction increased *C. elegans* thermotolerance and lifespan. In general, these findings suggested that anthocyanins themselves could not have a strong effect on improving *C. elegans* resistance against oxidative stress, which might support the discreet results obtained in the present study, where an extract mainly consisting of anthocyanins was assayed. It is difficult, however, to compare the results of these studies, as extracts of different origin and composition were used in each case, and distinct polyphenol and anthocyanin concentrations were assayed. Indeed, as commented above, the extracts assayed by other authors showed different anthocyanin profiles to ours, were mostly based on cyanidin and could also contain non-anthocyanin compounds. The extract used in the present work consisted of anthocyanins only, and its composition was complex, with up to 17 compounds detected derived from five different aglycones. These differences could explain the diverse behaviours observed in the different studies.

Regarding ROS, many authors have corroborated a decrease in their levels, under normal or oxidative stress conditions, after the treatment of *C. elegans* with anthocyanin-rich extracts from purple wheat [40], açai [41] or pitanga fruit [42]. In general, this decrease was indicated by the authors as one of the reasons to explain either the enhanced lifespan or the better survival rate of the worms under stress conditions.

Considering the results obtained herein, it is worth noting the distinct response of the nematodes against thermal stress, whatever their age, at each of the assayed concentrations (5 or 10 μg/mL) of the bilberry extract, highlighting the relevance of the dose in the produced effects. Actually, a reverse response seems to occur: while a mild protection of the worms against thermal stress is observed at the lowest concentration, detrimental effects are produced at the highest one. A similar behaviour has been previously described for polyphenols and other xenobiotics and falls within the concept of “hormesis”, a dose–response phenomenon characterised by the inversion of the response between low and high doses of a chemical, biological or physical agent (i.e., a J-shaped dose-response curve) [57,58,59,60]. Flavonoids, including anthocyanins, are an example of compounds that can display a hormetic behaviour [61]; indeed, although they are usually recognised as antioxidants and free radical scavengers, they are also able to exert in vivo pro-oxidant effects as a function of their concentration and the biological system [53].

Concerning the mechanisms of action involved in the effects of the anthocyanins at molecular level, some authors have proposed that they may interact with the insulin/IGF-1 signalling pathway (IIS), promoting the translocation of the transcription factor DAF-16, an orthologue of human FoxO, to the nucleus [39,40,41,42,43]. Another transcription factor that acts downstream of the IIS pathway is the heat shock factor 1 (HSF-1). When translocated to the nucleus, HSF-1 binds to DNA specific regions that contain heat shock elements (HSE), resulting in the induction of genes codifying molecular chaperones, which are known to be involved in longevity and thermo-tolerance in *C. elegans*. Thermal stress, for instance, is able to increase the levels of hsp-16.2 [62,63]. In addition to chaperones, both DAF-16/FoxO and HSF-1 regulate other stress response genes, such as catalase (*ctl-1*), superoxide dismutase-3 (sod-3), bacterial pathogen defence genes (*lys-7, spp-1*) or glutathione S-transferase (*gst-4*) [64].

**Figure 6:**
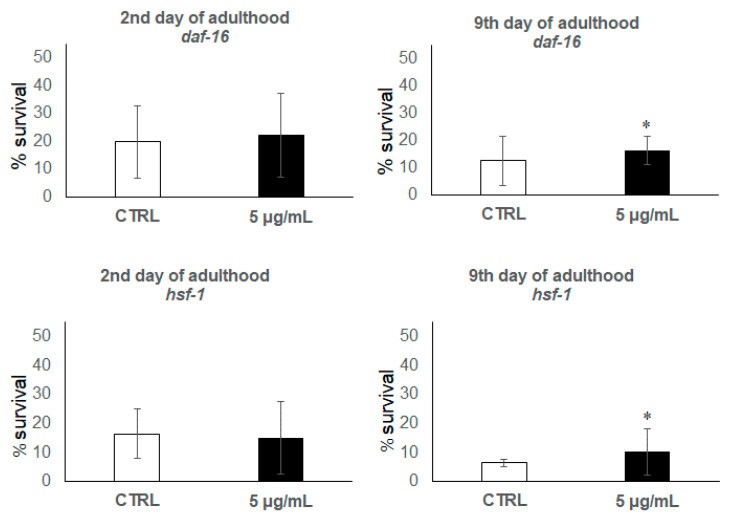
Percentages of worm survival at the 2nd and 9th day of adulthood after the application of thermal stress (35 °C, 8 h) in the *C. elegans* mutant strains *daf-16* and *hsf-1* grown in the absence (controls) and presence of 5 μg/mL of the bilberry anthocyanin extract. The results are presented as the mean values ± SD. The asterisk indicates a significant difference in relation to controls at *p* < 0.05.

In order to help to elucidate if these factors could be involved in the observed effects of the anthocyanin extract on *C. elegans* thermotolerance, assays were also carried out using the loss-of-function mutant strains *daf-16* and *hsf-1*. As for the wild-type N2 strain, mutant worms were subjected to a thermal shock (35 °C, 8 h) at the 2nd and 9th day of adulthood after being grown in the presence of the lowest anthocyanin concentration (5 μg/mL), for which positive effects on improving resistance against thermal stress were observed in the previous assays. As shown in Figure 6, the treatment with the bilberry extract did not produce a clear rise in the survival rate of any mutant in young worms (2nd day of adulthood). However, a significant increase in the resistance to thermal stress compared to the control was noticed in older worms (9th day). These results suggest that the intervention of both transcription factors can explain the effects of the anthocyanin-rich extract on resistance to stress in young individuals, whereas for worms in the post-reproductive development stage, the effect would be either independent of these genes or at least the presence of only one of them was sufficient or other compensatory mechanisms could be activated to compensate thermal stress.

These observations of the different implication of genes related to the IIS pathway depending on the age of the worms might explain some of the divergences found in the literature regarding the effects of anthocyanins/polyphenols on the longevity and stress resistance of *C. elegans*. Wilson et al. [43], working with worms at the 5th day of adulthood (post-reproductive period), found that blueberry polyphenols increased thermo-tolerance by acting independently of *daf-16* and *skn-1* genes, which is in agreement with our findings. SKN-1 is another transcription factor acting downstream of the IIS pathway homologue of the mammalian nuclear factor erythroid-2-related factor 2 (Nrf2), also involved in functions of cell protection [65]. However, when the assays have been conducted in larval stages or at the 1st day of adulthood, most authors have observed the implication of the DAF-16 transcription factor in the anthocyanins’ effects. Thus, Tambara et al. [42] showed that an extract from purple pitanga fruit administered to L1 larvae improved the survival of the worms after heat shock and demonstrated that the effect was dependent on DAF-16, as the extract did not provide protection in *daf-16* mutants. Similar conclusions have been drawn by Peixoto et al. [41], who checked the ability of an anthocyanin-rich extract from açai to reduce oxidative stress in worms at the L4 larval stage, confirming the translocation of DAF-16 to the nucleus, and by Chen et al. [40], who used L2 worms treated with purple wheat.

The relevance of the obtained results as related to human health is difficult to ascertain. In previous studies of our group [33,34,49], it was shown that the worm efficiently takes up and metabolises polyphenols, generating metabolites similar to those found in humans, thus constituting a suitable model for their study also considering some bioavailability issues. However, it is unknown how much of the compounds present in the culture media is taken in by the worm and which concentrations they can reach in the organism, so it is not possible to compare with those that could be present in human fluids and tissues after fruit or extract consumption. In this respect, *C. elegans* must be seen mostly as a model to delve into the mechanisms subjacent to the biological activity of polyphenols/anthocyanins, which seem to clearly not only rely on their direct antioxidant and radical scavenging capacities, as classically proposed. Another relevant implication of the obtained results is the notion that a hormetic response to anthocyanin intake can exist, which highlights the need to be careful about the consumption of supplements or highly enriched products.

## 4. Conclusions

Treatment with an anthocyanin bilberry extract at 5 μg/mL in the culture medium showed some protective effects on the modulation of ROS levels and stress resistance in *C. elegans*. A non-significant slight increase in their resistance against stress was induced in both young and older adults (2nd and 9th day of adulthood, respectively), as well as a significant decrease in ROS levels both in stressed and non-stressed aged worms. However, detrimental effects were observed in nematodes treated with the same bilberry extract at 10 μg/mL, leading to a higher worm mortality rate compared to the control. At this concentration, the treatment with the extract significantly increased ROS levels in young worms (2nd day), while decreasing them, though not significantly, in aged worms (9th day). This contradictory response exerted by the bilberry extract might be explained in view of the hormesis concept, which refers to a reverse response to the same exogenous substance when it is administered in low or high doses.

Taking into account the findings regarding ROS levels, the observations made in this study suggest that the effects of the bilberry extract on improving the resistance to stress may not only be due to its antioxidant capacity and direct radical scavenging activity, but it may also be mediated by other mechanisms, such as those related to biological plasticity, which might involve the selection of the phenotypes better adapted to external insults. The ability to modulate signalling pathways involved in worm survival, such as the evolutionary conserved insulin/IGF-1 signalling (IIS) pathway, could also play a crucial role. Assays in *C. elegans* mutant strains showed that the ability to modulate the transcription factors DAF-16 and HSF-1 that act downstream of the IIS pathway could be behind the improvement in the resistance against thermal stress produced by bilberry anthocyanins in young individuals, whereas they would not explain the effects produced in worms in the post-reproductive development stage, also supporting the existence of different compensatory mechanisms depending on worm age. Further experiments are, however, necessary to deeply investigate the actual mechanisms behind the biological effects of bilberry anthocyanins on the antioxidant response in living organisms, as well as to establish with certainty whether that response falls within the hormesis concept or not.

## Figures and Tables

**Table 1:** Content of anthocyanins in the lyophilised bilberry extract expressed as mg/g (delphinidin 3-*O*-glucoside equivalents) of dry extract.

Peak Number	Compound	mg/g of Extract
1	Delphinidin 3-*O*-galactoside	56.8
2	Delphinidin 3-*O*-glucoside	70.1
3	Cyanidin 3-*O*-galactoside	27.1
4	Delphinidin 3-*O*-arabinoside	47.8
5	Cyanidin 3-*O*-glucoside	37.3
6	Petunidin 3-*O*-galactoside	22.9
7	Cyanidin 3-*O*-arabinoside	41.5
8	Petunidin 3-*O*-glucoside	27.5
9	Peonidin 3-*O*-galactoside + Petunidin 3-*O*-arabinoside	17.5
10	Peonidin 3-*O*-glucoside + Malvidin 3-*O*-galactoside	34.7
11	Malvidin 3-*O*-glucoside	65.0
12	Malvidin 3-*O*-arabinoside	8.8
13	Cyanidin	10.9
14	Petunidin	5.7
15	Malvidin	5.3
	Total anthocyanins	478.9
